# Willingness of Greek general population to get a COVID-19 vaccine

**DOI:** 10.1186/s41256-021-00188-1

**Published:** 2021-01-29

**Authors:** Georgia Kourlaba, Eleni Kourkouni, Stefania Maistreli, Christina-Grammatiki Tsopela, Nafsika-Maria Molocha, Christos Triantafyllou, Markela Koniordou, Ioannis Kopsidas, Evangelia Chorianopoulou, Stefania Maroudi-Manta, Dimitrios Filippou, Theoklis E. Zaoutis

**Affiliations:** 1 Center for Clinical Epidemiology and Outcomes Research (CLEO), Athens, Greece; 2 ECONCARE LP, Athens, Greece; 3 Department of Anatomy and Surgical Anatomy, Medical School, National and Kapodistrian University of Athens, Athens, Greece; 4 Division of Infectious Diseases, The Children’s Hospital of Philadelphia, Perelman School of Medicine at the University of Pennsylvania, Philadelphia, USA

**Keywords:** Vaccine hesitancy, COVID-19, Europe, Vaccine coverage

## Abstract

**Background:**

Epidemiological data indicate that a large part of population needs to be vaccinated to achieve herd immunity. Hence, it is of high importance for public health officials to know whether people are going to get vaccinated for COVID-19. The objective of the present study was to examine the willingness of adult residents in Greece to receive a COVID-19 vaccine.

**Methods:**

A cross-sectional was survey conducted among the adult general population of Greece between April 28, 2020 to May 03, 2020 (last week of lockdown), using a mixed methodology for data collection: Computer Assisted Telephone Interviewing (CATI) and Computer Assisted web Interviewing (CAWI). Using a sample size calculator, the target sample size was found to be around 1000 respondents. To ensure a nationally representative sample of the urban/rural population according to the Greek census 2011, a proportionate stratified by region systematic sampling procedure was used to recruit particpants. Data collection was guided through a structured questionnaire. Regarding willingness to COVID-19 vaccination, participants were asked to answer the following question: “If there was a vaccine available for the novel coronavirus, would you do it?**”**

**Results:**

Of 1004 respondents only 57.7% stated that they are going to get vaccinated for COVID-19. Respondents aged > 65 years old, those who either themselves or a member of their household belonged to a vulnerable group, those believing that the COVID-19 virus was not developed in laboratories by humans, those believing that coronavirus is far more contagious and lethal compared to the H1N1 virus, and those believing that next waves are coming were statistically significantly more likely to be willing to get a COVID-19 vaccine. Higher knowledge score regarding symptoms, transmission routes and prevention and control measures against COVID-19 was significantly associated with higher willingness of respondents to get vaccinated.

**Conclusion:**

A significant proportion of individuals in the general population are unwilling to receive a COVID-19 vaccine, stressing the need for public health officials to take immediate awareness-raising measures.

**Supplementary Information:**

The online version contains supplementary material available at 10.1186/s41256-021-00188-1.

## Background

On March 11, 2020, World Health Organization (WHO) described the spread of corona-virus disease 2019 (COVID-19) as a pandemic. COVID-19, caused by severe acute respiratory syndrome corona-virus 2 (SARS-CoV-2). Currently scientists found that the virus stains in Italy and some other countries are different in Wuhan, and later on these virus strains were found existing earlier before the virus detected in Wuhan, suggesting possible multi-point transmission of the SARS-CoV-2 [1, 2]. COVID-19 has expanded internationally reaching 66,243,918 confirmed cases and 1,528,984 deaths worldwide by December 8th, 2020 [3]. Beyond the health crisis, the outbreak of the pandemic has disrupted the financial and social systems of most countries increasing poverty and wealth inequality [4, 5].

Greece reported its first confirmed case on February 26th, 2020 [6]. Up until December 8th, 116,721 people have been diagnosed with COVID-19 and 3092 have died [6]. The Greek Government preemptively announced a series of strict measures, including the early adoption of a lockdown (in mid-March), and immediately started a nation-wide public information campaign regarding COVID–19 prevention emphasizing the importance of adopting disease control measures during the lockdown in order to limit the spread of COVID-19 [7]. These measures led Greece to the lowest number of 30-day mortality per million population after Norway and Finland. In contrast, other Southern Europe countries with similar statistics to Greece regarding Gross Domestic Health Expenditure and age distribution, but which delayed initiating lockdown, suffered some of the highest losses from the pandemic [8].

To curtail the spread of the novel corona-virus, pharmaceutical companies and academic institutions globally are working to develop a vaccine. According to the WHO, nearly 180 vaccine candidates are being tested around the world, but none has yet completed clinical trials, at the time of writing. Just one vaccine developed by the Gamaleya Research Institute in Moscow was approved by the Ministry of Health of the Russian Federation on August 11th but it has not launched Phase III of clinical trial [9, 10]. Researchers estimate that a vaccine is likely to become widely available by mid-21, approximately 12–18 months after SARS-CoV-2 first appeared [10].

It is widely known that the effectiveness of a COVID-19 vaccine depends on adequate uptake [11, 12]. Based on the currently available epidemiological data, SARS-CoV-2 is a highly transmissible virus and in order to break the chain of transmission, at least 55–82% of the population need to be vaccinated to achieve herd immunity [13]. However, there is a growing body of evidence declaring that even nowadays, low vaccination rates have remained an issue of concern while vaccine hesitancy has become more prevalent [14, 15]. Compliance with the anti-H1N1 vaccine during the 2009 influenza pandemic was very low worldwide and particularly in Europe [16, 17]. The WHO has named vaccine hesitancy as one of the top 10 threats to global health, in 2019 [18]. As such, the next challenge that public health officials will have to overcome regarding COVID-19 is to achieve a high vaccination rate among the public. For this reason, it is important to investigate whether or not people are willing to get vaccinated.

To the best of our knowledge, limited data are available in the international literature regarding the willingness of public to receive a COVID-19 vaccine [12, 13, 19–22]. A multi-country European study showed that 73.9% of the participants would be willing to get vaccinated against COVID-19 if a vaccine would be available [12]. Greece, which managed to keep COVID*-*19 deaths incredibly low and presented as a success story worldwide, did not participate in this European study and as such no data are available regarding the willingness of Greek adult population to be vaccinated.

Hence, the objectives of this study were to: (1) examine the extent to which adults in Greece are willing to receive a COVID-19 vaccine if one was to become available and (2) determine whether socio-demographic, clinical factors as well as knowledge, attitudes and practices (KAP) of adult population against COVID-19 are associated with their willingness.

## Methods

### Study design and data collection

A nationwide cross-sectional survey was conducted in Greece, between April 28, 2020 to May 03, 2020 (last week of lockdown) with a primary objective of assessing the knowledge, attitudes and practices (KAP) of adult population against COVID-19, using a mixed methodology for data collection: Computer Assisted Telephone Interviewing (CATI) and Computer Assisted web Interviewing (CAWI). To calculate the sample size for this survey, we hypothesized that at a 99% confidence interval, 50% of the respondents would have a satisfactory knowledge level of COVID-19 at a margin of error ± 5%. Using a sample size calculator, the target sample size was found to be around 1000 respondents. To ensure a nationally representative sample of the urban/rural population according to the Greek census 2011 (www.statistics.gr), a proportionate stratified by region random sampling procedure was used to recruit participants. Half of the total target sample size was met by CATI (502 interviews) and the other half (502 interviews) by CAWI. For CATI, a random-digit dialled (RDD) sample of 9977 landline-telephone households was conducted, 74% of whom were excluded due to unavailability (e.g. no answer, busy etc.) but 26% of whom (2597) led to handled calls. Of those 2597, 1434 refused to participate in this survey, 50 asked for the interview to be terminated before screening was complete, 219 did not meet an age criterion, 228 calls were reached businesses/commercial spaces instead of housholds and 142 communications were not possible. To complete 502 interviews with the CAWI method, 1497 invitations were sent to adults. The response rate for CATI method was 25%, and for CAWI method 33.5%.

To ensure the external validity and the greater generalizability of the study, survey weights were used to adjust for differences in age and gender distribution between survey sample and country population as obtained from the census 2011 (www.statistics.gr). The survey weights were calculated with the rake method (also known as “rim”).

Prior to completion of the survey, an informed oral consent procedure was followed. The study protocol was not submitted to the ethical committee of any institution for approval, since according to the Greek legislation (Law 2328/1995, Presidential Decree 310/1996, Law 3603/2007, Law 2472/1997, Law 3471/2006) there is no need for ethics approval in telephone and internet surveys such as the one presented here (Association of Opinion Polls and Survey Organizations - www.sedea.gr).

### Questionnaire development

A structured questionnaire was developed in the Greek language to collect data [see Additional file 1]. This questionnaire consisted of four main themes, as follows: 1) demographics, which surveyed participants’ socio-demographic information; 2) knowledge about COVID-19; 3) attitudes toward COVID-19; and 4) practices to control COVID-19. The survey took 9–12 min to be completed. The questionnaire was pre-validated by three independent reviewers, and a pre-test study was conducted with 6 individuals. The responses from the pre-test were not included in the analyzed data.

To measure knowledge about COVID-19, 23 questions were used. A total knowledge score ranging from 0 to 23 was calculated assigning 1 point to each correct answer. The answers considered correct per question are presented in additional file 2. Higher score indicating better knowledge of COVID-19. The internal consistency of the questions used in the total score was assessed using Cronbach’s alpha coefficient that was found to be 0.580, indicating “poor” internal reliability. Participants were also asked to state how they received information about COVID-19.

Attitudes towards COVID-19 were measured through 8 questions and practices relevant to COVID-19 control were measured through 7 questions. Among the practices assessed was the willingness of respondents to receive a COVID-19 vaccine when it is to be available. To be more specific, participants were asked to answer the following question: “If there was a vaccine available for the novel coronavirus, would you do it?”, and the three potential answers were “yes”, “no” and “not sure”.

### Statistical analysis

Participant’s responses are presented with absolute and relative frequencies (%), whereas knowledge score is presented with median and interquartile range (IQR). Chi-square test of independence was applied to identify possible factors (demographics, knowledge, attitudes and practices against coronavirus) associated with participant’s intension to be vaccinated against COVID-19 if a vaccine was available. Multiple logistic (stepwise) regression was performed to identify independently associated factors with the willingness of Greek adult population to be vaccinated against COVID-19. Factors found to be significantly associated with willingness at a univariate level were entered in a multiple logistic regression. In case of factors found to be strongly correlated (i.e. age and vulnerable group), one of these were selected to be entered in the multiple logistic regression model to avoid collinearity issues. Results are presented with Odds Ratios (OR) and 95% Confidence intervals (CI). The level of statistical significance was set to 5%. Analysis was conducted with SPSS statistical package v.25.

## Results

### Characteristics of participants

In total of 1004 respondents recruited in the survey, the mean age was 41.7 years (SD: 17.7). Of all respondents, 51.0% were female, 59.8% had received college or above education, 63.8% were married or cohabiting, and 52.0% reported that they worked before the COVID measures were taken. Almost 35.0% of respondents reported that they belong to a vulnerable group and 41.2% reported that they have a member of their household belonging to a vulnerable group (Table 1).

**Table 1:** Associations between demographic characteristics and willingness of respondents to get vaccinated for COVID-19 in Greece

		Willingness to be vaccinated against Coronavirus if a vaccine was to become available			
	Total (***N*** = 1004) n (%)	No/Don’t Know (***N*** = 425) n (%)	Yes (***N*** = 579) n (%)	***p***-value	OR (95% CI) ^a^
**Gender**				0.104	
Male	512 (51.0%)	204 (39.9%)	308 (60.1%)		1.00
Female	492 (49.0%)	221 (44.9%)	271 (55.1%)		0.82 (0.63–1.05)
**Age (years)**				**0.001**	
18–24	109 (10.9%)	49 (45.2%)	60 (54.8%)		1.00
25–34	170 (16.9%)	88 (52.0%)	82 (48.0%)		0.76 (0.47–1.24)
35–44	185 (18.4%)	96 (51.9%)	89 (48.1%)		0.76 (0.48–1.23)
45–54	166 (16.5%)	80 (48.3%)	86 (51.7%)		0.88 (0.54–1.43)
55–64	142 (14.1%)	63 (44.5%)	79 (55.5%)		1.03 (0.62–1.70)
65+	232 (23.1%)	48 (20.7%)	184 (79.3%)		3.16 (1.93–5.17)*
**Age (years)**				**0.001**	
< 65	772 (76.9%)	377 (48.8%)	395 (51.2%)		1.00
65+	232 (23.1%)	48 (20.7%)	184 (79.3%)		3.65 (2.57–5.17)*
**Residence**				0.876	
Athens	353 (35.2%)	144 (40.7%)	210 (59.3%)		1.00
Thessaloniki	138 (13.7%)	60 (43.5%)	78 (56.5%)		0.89 (0.60–1.33)
Urban area (> 10.000 inhabitants)	328 (32.6%)	141 (42.9%)	187 (57.1%)		0.91 (0.67–1.24)
Semi-urban or Agricultural area	185 (18.4%)	81 (43.6%)	104 (56.4%)		0.89 (0.62–1.28)
**Education Level** ^b^				0.082	
Primary school	68 (6.8%)	20 (29.2%)	48 (70.8%)		1.00
Middle school	335 (33.4%)	146 (43.6%)	189 (56.4%)		0.54 (0.30–0.94)*
College and above	601 (59.8%)	259 (43.1%)	342 (56.9%)		0.54 (0.32–0.94)*
**Marital Status**				0.053	
Single	268 (26.9%)	129 (48.0%)	140 (52.0%)		1.00
Married/ Cohabitation	636 (63.8%)	257 (40.5%)	379 (59.5%)		1.36 (1.02–1.81)*
Divorced/Widowed	93 (9.3%)	34 (36.0%)	60 (64.0%)		1.64 (1.01–2.67)*
**Children**				**0.008**	
No	682 (68.4%)	269 (39.4%)	413 (60.6%)		1.00
Yes	315 (31.6%)	152 (48.4%)	162 (51.6%)		0.69 (0.53–0.91)*
**Do you personally belong to a vulnerable group?**				**0.001**	
No	648 (65.2%)	315 (48.6%)	333 (51.4%)		1.00
Yes	346 (34.8%)	100 (28.8%)	246 (71.2%)		2.34 (1.78–3.09)*
**Living with someone belonging to a vulnerable group?**				**0.010**	
No	585 (58.8%)	265 (45.3%)	320 (54.7%)		1.00
Yes	410 (41.2%)	152 (37.1%)	258 (62.9%)		1.40 (1.08–1.84)*
**Work before coronavirus outbreak**				**0.001**	
No	479 (48.0%)	161 (33.5%)	319 (66.5%)		1.00
Yes	519 (52.0%)	259 (49.9%)	260 (50.1%)		0.51 (0.39–0.65)*

*OR* Odds Ratio, *CI* Confidence Interval

* p < 0.05

^a^ Results from univariate logistic regression models

^b^ **Primary school**, a school for children between the ages of about five and eleven; **Middle school**, is the next step up from primary school (ages 12 to 18)

### Factors associated with willingness to receive a COVID-19 vaccine

In total, 57.7% (*n* = 579) of respondents stated that they are going to get vaccinated for COVID-19 when a vaccine is available, while 26.0% (*n* = 261) stated that they are unwilling to receive a COVID-19 vaccine and 16.3% (*n* = 164) named that they are unsure.

Willingness to receive a COVID-19 vaccine by socio-demographic characteristics is presented in Table 1. It was found that respondents aged more than 65 years old [OR (95% CI): 3.65 (2.57–5.17)], married/cohabiting [OR (95% CI): 1.36 (1.02–1.81)] and divorced/widowed [OR (95% CI): 1.64 (1.01–2.67)], those who did not work before lock-down [OR (95% CI): 1.96 (1,54–2.56)], those who belonged to a vulnerable group [OR (95% CI): 2.34 (1.78–3.09)], those who have a member of their household belonging to a vulnerable group [OR (95% CI): 1.40 (1.08–1.84)] and those with no children [OR (95% CI): 1.45 (1.09–1.89)] over their counterparts were statistically significantly more likely than their counterparts to be willing get a future COVID-19 vaccine.

**Fig. 1:**
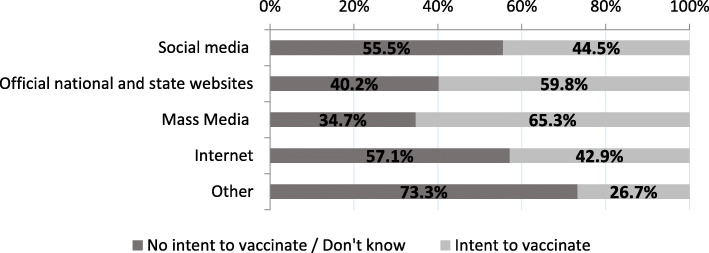
Association between source of information regarding COVID-19 and willingness of respondents to get vaccinated in greece

Table 2 presents the association between knowledge of respondents regarding symptoms, transmission routes and prevention and control measures against COVID-19 and their willingness to get vaccinated when the vaccine is to be available. Respondents reported correctly the 5 most common symptoms of COVID-19 [OR (95% CI): 1.54 (1.18–2.02)], those provided the correct answer in all questions regarding the transmission routes [OR (95% CI): 1.43 (1.04–1.96)] and those found to know the appropriate control and prevention measures [OR (95% CI): 1.62 (1.13–2.32)] as well as those knowing the appropriate way of handwashing with soap and water [OR (95% CI): 1.39 (1.08–1.80)] were statistically significantly more likely by 54, 43, 62 and 39%, respectively, to be willing to get vaccinated for COVID-19 (Table 2). Moreover, it was found that the source used by respondents to get informed regarding COVID-19 was significantly associated with their willingness to receive a COVID-19 vaccine (*p* < 0.001). More specifically, respondents informed by mass media and official national and state websites were more likely to be willing to get vaccinated over those informed by social media, internet, or other sources (Fig. 1).

**Table 2:** Associations between knowledge against Corona Virus and willingness of respondents to get vaccinated for COVID-19 in Greece

		Willingness to be vaccinated against Coronavirus if a vaccine was to become available			
	Total (***N*** = 1004) n (%)	No/Don’t Know (***N*** = 425) n (%)	Yes (***N*** = 579) n (%)	***p***-value	OR (95% CI)^**a**^
**Knowledge of symptoms** ^**b**^				**0.001**	
No	652 (65.0%)	300 (46.0%)	352 (54.0%)		1.00
Yes	352 (35.0%)	125 (35.6%)	227 (64.4%)		1.54 (1.18–2.02)*
**Knowledge of transmission routes**^**b**^				**0.025**	
No	798 (79.4%)	352 (44.1%)	446 (55.9%)		1.00
Yes	206 (20.6%)	73 (35.6%)	133 (64.4%)		1.43 (1.04–1.96)*
**Knowledge of prevention measures**^**b**^				**0.007**	
No	846 (84.2%)	373 (44.1%)	472 (55.9%)		1.00
Yes	158 (15.8%)	52 (32.8%)	107 (67.2%)		1.62 (1.13–2.32)*
**Knowledge of correct first action in case of COVID-19 related symptoms** ^**b**^				0.237	
No	192 (19.3%)	88 (45.7%)	104 (54.3%)		1.00
Yes	802 (80.7%)	330 (41.1%)	472 (58.9%)		1.20 (0.88–1.65)
**Knowledge of the appropriate way of hand washing with soap and water**^**b**^				**0.012**	
No	602 (60.0%)	274 (45.6%)	328 (54.4%)		1.00
Yes	402 (40.0%)	151 (37.5%)	251 (62.5%)		1.39 (1.08–1.80)*
**Is handwashing with antiseptic/alcoholic solution better than soap and water?**				0.480	
No	807 (80.3%)	346 (42.8%)	461 (57.2%)		1.00
Yes	197 (19.7%)	79 (40.3%)	118 (59.7%)		1.11 (0.81–1.53)
	**Median (IQR)**	**Median (IQR)**	**Median (IQR)**		
**Total Knowledge Score**^**b**^	17 (16–19)	17 (15–18)	18 (16–19)	**0.001**	1.16 (1.10–1.22)*

*OR* Odds Ratio, *CI* Confidence Interval, *IQR* Interquartile range

* p < 0.05

^a^ Results from univariate logistic regression models

^b^ As defined in Additional file 2

Table 3 presents the association between the attitudes and practices of the Greek adult population regarding COVID-19 and their willingness to get vaccinated for COVID-19 when a vaccine is to be available. It was found that respondents believing that the COVID-19 virus was not developed in laboratories by humans [OR (95% CI): 4.00 (2.86–5.56)], those believing that coronavirus is far more contagious [OR (95% CI): 2.50 (1.81–3.44)] and lethal compared to the H1N1 virus [OR (95% CI): 2.73 (2.05–3.62)], those believing that next waves are coming [OR (95% CI): 1.66 (1.25–2.21)] and those not believing that the spread of COVID-19 will be eliminated when a large percentage of the population has become infected [OR (95% CI): 3.57 (2.56–5.00)] were statistically significantly more likely to be willing to receive a COVID-19 vaccine compared to their counterparts. Moreover, respondents who had been vaccinated for seasonal flu were significantly more likely to be willing to get vaccinated for COVID-19 [OR (95% CI): 4.21 (3.09–5.73)] (Table 3).

**Table 3:** Associations between attitudes & practices and willingness of respondents to get vaccinated for COVID-19 in Greece

		Willingness to be vaccinated against Coronavirus if a vaccine was available			
	Total (***N*** = 1004) n (%)	No/Don’t Know (***N*** = 425) n (%)	Yes (***N*** = 579) n (%)	***p***-value	OR (95% CI) ^*a*^
**Do you believe that the novel coronavirus was developed by humans in laboratories?**				**0.001**	
No	307 (30.6%)	74 (24.0%)	234 (76.0%)		1.00
Yes	458 (45.6%)	255 (55.6%)	203 (44.4%)		0.25 (0.18–0.35)*
Don’t Know	239 (23.8%)	96 (40.4%)	142 (59.6%)		0.47 (0.32–0.67)*
**Coronavirus far more infectious compared to the flu virus H1N1**				**0.001**	
No	196 (20.6%)	117 (59.8%)	79 (40.2%)		1.00
Yes	757 (79.4%)	283 (37.4%)	474 (62.6%)		2.50 (1.81–3.44)*
**Coronavirus far more lethal compared to the flu virus H1N1**				**0.001**	
No	290 (31.0%)	170 (58.5%)	120 (41.5%)		1.00
Yes	647 (69.0%)	220 (34.1%)	426 (65.9%)		2.73 (2.05–3.62)*
**Do you think that the spread of the novel coronavirus will be mitigated when a large percentage of the population has become infected?**				**0.001**	
No	734 (80.0%)	244 (33.2%)	490 (66.8%)		1.00
Yes	183 (20.0%)	118 (64.3%)	65 (35.7%)		0.28 (0.20–0.39)*
**Very likely to have other waves of coronavirus outbreaks in our country**				**0.001**	
No	261 (27.1%)	133 (51.0%)	128 (49.0%)		1.00
Yes	702 (72.9%)	271 (38.5%)	431 (61.5%)		1.66 (1.25–2.21)*
**Where you in the process of social distancing before the government measures were applied?**				0.065	
No	573 (58.2%)	252 (44.0%)	321 (56.0%)		1.00
Yes	412 (41.8%)	157 (38.1%)	255 (61.9%)		1.27 (0.98–1.65)
**In average how many times do you wash your hands on a daily basis?**				0.160	
< 10 times	582 (58.7%)	256 (44.0%)	326 (56.0%)		1.00
≥ 10 times	410 (41.3%)	162 (39.5%)	248 (60.5%)		1.20 (0.93–1.55)
**Did you do the seasonal flu vaccine this year?**				**0.001**	
No	665 (67.1%)	349 (52.4%)	317 (47.6%)		1.00
Yes	327 (32.9%)	68 (20.7%)	259 (79.3%)		4.21 (3.09–5.73)*

*OR* Odds Ratio, *CI* Confidence Interval

* p < 0.05

^a^ Results from univariate logistic regression models

Multiple logistic regression model (Table 4) revealed that those belonging to a vulnerable group were 37% more likely to get vaccinated for COVID-19 than those who were not [OR (95% CI): 1.37(1.01–1.94)]. Those who were working before coronavirus outbreak were 38% less likely to get vaccinated for COVID-19 than those who were not [OR (95% CI): 0.62(0.44–0.89)]. One-point increase in participant’s knowledge score indicated a 12% increase in the willingness of respondents to receive the COVID-19 vaccine [OR (95% CI): 1.12(1.05–1.20)]. Those believing that the novel coronavirus is more lethal than flu and those believing that a new outbreak is coming were 70 and 49% more likely, respectively, to get vaccinated for COVID-19 [OR (95% CI): 1.70(1.19–2.44) and OR (95% CI): 1.49(1.03–2.17), respectively]. On the contrary, those believing that the coronavirus was developed by humans in laboratories and those believing that coronavirus will be eliminated when a large percentage of the population will be infected were 73 and 66% less likely, respectively, to be willing to get vaccinated for COVID-19 [OR (95% CI): 0.27(0.18–0.39) and OR (95% CI): 0.34(0.23–0.51), respectively].

**Table 4:** Impact of factors on participant’s intension to vaccinate against Coronavirus

	OR (95% CI)	***p***-value
**Enter Logistic Regression**		
**Education Level (Reference: Primary school)**		
Middle school	0.63 (0.29–1.35)	0.232
College and above	0.64 (0.30–1.37)	0.249
**Marital Status (Reference: Single)**		
Married/ Cohabitation	1.04 (0.70–1.57)	0.833
Divorced/Widowed	1.12 (0.58–2.16)	0.735
**Having Children**	0.88 (0.61–1.28)	0.513
**Belonging to a vulnerable group**	1.37 (1.01–1.94)	**0.047**
**Working before coronavirus outbreak**	0.62 (0.44–0.89)	**0.010**
**Knowledge Score**^**a**^	1.12 (1.05–1.20)	**0.001**
**Source of Info (Reference: Social Media)**		
Official national and state websites	1.45 (0.81–2.59)	0.214
Mass Media	1.46 (0.86–2.48)	0.156
Internet	0.65 (0.34–1.24)	0.189
**Belief that coronavirus was developed by humans in laboratories (Reference: No)**		
Yes	0.27 (0.18–0.39)	**0.001**
Don’t Know	0.40 (0.25–0.65)	**0.001**
**Belief that coronavirus far more lethal compared to the flu virus H1N1**	1.70 (1.19–2.44)	**0.004**
**Belief that coronavirus will be eliminated when a large percentage of the population will be infected**	0.34 (0.23–0.51)	**0.001**
**Belief that new coronavirus outbreak is very likely**	1.49 (1.03–2.17)	**0.036**

*OR* Odds Ratio, *CI* Confidence Interval

^a^ As defined in Additional file 2

A sensitivity analysis was also conducted adding respondent’s that were unsure about receiving a COVID-19 vaccine with those that were positive. No significant differences regarding the aforementioned results were detected.

## Discussion

In the race to stop the novel coronavirus that has killed more than half a million people and crippled economies across the globe, many companies and academic institutions are working to develop a vaccine. The effectiveness of the upcoming vaccine depends on the population coverage, since in the case of a low vaccination rate, herd immunity will not be developed, and the most vulnerable population groups will not be protected. As such, it is important to understand beforehand the public’s intention to get vaccinated for COVID-19, so that public health officials have the time to design and implement targeted interventions to raise the awareness of general population about the importance of vaccination. After all, hesitancy implies that minds can be swayed towards acceptance, if presented with the right information.

In this context, the objective of this nationwide cross-sectional survey conducted in Greece, was to assess the willingness of adult general population to receive a COVID-19 vaccine when it is to be available as well as factors that might affect their willingness. To the best of our knowledge, this study is the first one conducted in Greece and its results are expected to have a great impact on decisions taken by public health officials.

Our results revealed that over two out of five individuals were unwilling or unsure about receiving a COVID-19 vaccine and only 57.7% stated that they will get vaccinated. The percentage of individuals willing to get vaccinated is not large enough to achieve herd immunity either through vaccination or prior infection transmission based on the estimates of the basic reproduction number (R_0_) [23, 24]. Comparing the willingness rate of our study with that of similar studies conducted in the UK (76.9%) [13] or other European countries (ranged from 62 to 80%) [12], it seems that Greek people are more hesitant against COVID-19 vaccine compared to other European populations. A similar variability across countries had been observed in 2009 for the acceptance of H1N1 influenza pandemic vaccine with this rate ranging between 8 and 67% and with Greeks reporting lower acceptance rates (ranged from 9.1 to 22.9% depending on the week of the survey) compared to other European populations [25]. In case that willingness rates and as such vaccination rates remain low, pandemic will continue to have negative effects on population’s health and country’s economy. In this context, there is an urgent need for an awareness campaign to be designed and implemented by Greek public health officials aiming to increase acceptance rates for COVID-19 vaccine by the Greek general population.

Vaccine hesitancy is a multifaceted, complex issue rooted in multiple values: particularly liberty, risk perception, and distrust. The hesitancy of general population against COVID-19 vaccine could be explained by concerns about the safety and effectiveness of the vaccine or belief that the individuals are not at risk of becoming ill, reasons provided by Greek people to justify their hesitancy over H1N1 flu vaccine [17]. Available data from the previous pandemic of H1N1 indicate that the actual intention of vaccination could be different when a vaccine is available [26]. It is remarkable that in 2009 only a small percentage of approximately 3% of the entire Greek population - ended up receiving a vaccination by the end of December 2009 (4 months after the release), one of the lowest in the European Union [27]. Fortunately, studies have shown that vaccination coverage rates increased over time [28].

Drivers of vaccine hesitancy rely heavily on both belief systems and personal experiences. Our results indicate that specifically socio-demographic categories and medical history affects the willingness to vaccinate for COVID-19 or not. It was found that people aged over than 65 years old or people who belonged to or had a household member belonging to a vulnerable group were statistically significantly more likely to be willing to have a COVID-19 vaccine. Our results are not in line with the findings of similar studies conducted in UK [13] and in France [29]. In the UK study, willingness was not affected by the increased risk of COVID-19 while in France people aged older than 75 years were reluctant to get vaccinated. Our study identified individuals with a lower educational background as the ones more likely to get vaccinated for COVID-19 while studies in UK [13] and Australia [19] demonstrated the opposite. The aforementioned findings indicate that younger people, individuals not belonging to vulnerable groups or not having a vulnerable family member and those with higher educational background could be a target population in educational campaigns about vaccine safety and efficacy since that are currently hesitant to get vaccinated.

Moreover, respondents that had not been vaccinated for seasonal flu and those believing that the coronavirus was man-made in a laboratory were significantly more likely to be negative towards getting vaccinated for COVID-19. This has been previously described by Neumann-Bohme et all [12] who identified concerns about side effects and safety of the vaccine, general rejection of vaccines and beliefs of conspiracy theories as reasons for not wanting to vaccinate. It has also been previously shown that people are more likely to reject new vaccines than familiar ones [30]. It is imperative that the above reasons are taken into consideration in local public health officials’ strategies and addressed explicitly in public communication campaigns.

Public awareness campaigns tailored to specific community needs have proven most effective in raising vaccination rates for other outbreaks [31]. Our study revealed that among Greek citizens more knowledgeable respondents on the virus’s transmission routes and prevention measures were more likely to get vaccinated. Regarding the source of information, respondents informed by social media, internet, or other sources were less willing to get vaccinated, indicating that social media can be a source of false information. Emerging evidence suggests that correcting misinformation on social media may be effective in changing health beliefs [32].

This study has both strengths and limitations. The strength of this study lies in its study design. In previous KAP studies, data collection was conducted using online self-reported questionnaires that have the disadvantage of limiting the participation of vulnerable groups, such as illiterate and rural people, without access to the internet and online health information resources. In our survey, a mixed methodology for data collection was used (CATI and CAWI) ensuring a random sample with greater generalizability in terms of age, gender and residence area. It should be mentioned that, according to the international bibliography, this is the first KAP study that conducted in Europe, contributing to the determination of the knowledge, perceptions and practices of the general population of a European country with different culture and way of life comparing with a country from another continent. Moreover, this is the first study in a nationally representative sample of the urban/rural population that demonstrated public’s willingness to receive COVID-19 vaccine if one was to become available.

Our study has also limitations. This was a cross sectional study so it depicts a picture of the community response at the point of the study. The real intention of vaccination against COVID-19 could be different when the vaccine is available. Secondly, as this study used self-reported data, it is possible that participants may have answered questions based on what they perceive to be expected of them (reporting bias) [33]. Furthermore, given that the primary objective of the present survey was to assess KAP of general population against COVID-19, we haven’t collected data regarding the reasons of acceptance, hesitancy and unwillingness to do the vaccine.

## Conclusions

In summary, almost half of adult population in Greece seems to be hesitant against the SARS-CoV-2 vaccine when it becomes available. Younger people, individuals not belonging to vulnerable groups or not having a vulnerable family member, those informed by social media, internet, or other non-official sources, people with the perception that the coronavirus was man-made in a laboratory could be potential target groups for interventions aiming to increase public’s awareness regarding vaccination. This study is expected to provide useful insights to government agencies, health care workers and other authorities to mitigate the impact of vaccine hesitancy. Further studies need to be conducted to assess the change in the public’s willingness to be vaccinated as clinical trials for vaccine development progress to completion.

## Supplementary Information

**Additional file 1.**

**Additional file 2.**

## Data Availability

The datasets used and/or analyzed during the current study are available from the corresponding author on reasonable request.

## Abbreviations

COVID-19: Coronavirus Disease 2019
CATI: Computer Assisted Telephone Interviewing
CAWI: Computer Assisted Web Interviewing
KAP: Knowledge, Attitudes, and Practices
IQR: interquartile range
OR: Odds Ratios
CI: Confidence interval
