# Supplementary material for: Willingness of Greek general population to get a COVID-19 vaccine
Source: Glob Health Res Policy. 2021 Jan 29;6:3. doi: 10.1186/s41256-021-00188-1 (PMC7843240; doi:10.1186/s41256-021-00188-1)
Supplement: Supplementary file 1 — Additional file 1. [file 41256_2021_188_MOESM1_ESM.docx]

**Additional file 1**

**Questionnaire-** **KNOWLEDGE, ATTITUDES AND PRACTICES (KAP) RELATED TO THE COVID-19 PANDEMIC AMONG THE GREEK GENERAL POPULATION**

Good morning. My name is . . . and I work for (name of company), an independent organization of (clinical) research. We are conducting a research study regarding the novel coronavirus pandemic.

**1.** First of all, I would like to ask if you are over the age of 18 years old?

Yes □ No □ I do not answer □

**If «NO» or «I DO NOT ANSWER»** -> Thank him/her and close the phone

**If «YES»** -> read the informed consent.

“We are conducting a study in order to assess the knowledge, attitudes and behaviors of the general public regarding the novel coronavirus pandemic. We would like to inform you that all of the data collected during this survey will be anonymous and it will not be possible to identify you individually from your answers. The practices/methods of this study are in compliance with the law, Acts and provisions of the national data protection authorities/ European Data Protection Supervisor (EDPS). Each of your answers will be presented together with/along with other participants’ answers. The duration of this survey will be a few minutes.

Taking into consideration all of the above would you like to participate in this study?”

□ Yes (Continue)

□ No (Thank him/her and close the phone)

| **Α. DEMOGRAPHIC CHARACTERISTICS** | |
| --- | --- |
| **Α1. Gender of Participant** | Male □ Female □ |
| **Α2. Age of Participant**  (Note specific age) _____ | □ 18 – 29  □ 30 – 39  □ 40 – 49  □ 50 – 59  □ 60 – 69  □ 70 – 79  □ 80+ |
| **Α3.** **Population wise in which of the following categories does your household belong?** | □ Athens  □ Thessaloniki  □ Urban area, apart from Athens and Thessaloniki (> 10.000 citizens/inhabitants)  □ Semi-urban  □ Agricultural area |
| **Α3Α. In which state/prefecture does your household belong?** | List of states/prefectures |
| **Α4. Which is the status of your education?** | □ Elementary school  □ Junior high  □ High school  □ Higher education  □ MSc and/or PhD student/holder |
| **Α5. Which of the following is your marital status?** | □ Single  □ Married  □ Divorced - Separated  □ Widower/ Widow  □ Cohabitation |
| **Α6. Do you have under aged children living at home with you?** | □ Yes  □ No |
| **Α7. If yes, how many?** | Define . . . |
| **Α8. How many people live in your household, including yourself?** | ……… |
| **Α9A. Do you personally belong to α vulnerable group?**  **(>65yo, pregnant, diabetes mellitus, chronic cardiovascular disease, chronic respiratory problems, malignancy, immunosuppression)** | □ Yes  □ No |
| **Α9Β. Do you have other people living with you who belong to a vulnerable group? (>65yo, pregnant, diabetes mellitus, chronic cardiovascular disease, chronic respiratory problems, malignancy, immunosuppression)** | □ Yes  □ No |
| **Α10.α. Before the government measures were taken, were you actively working?** | Yes □ No □ |
| **Α10.β If yes, is your occupation any of the following?** | □ Doctor  □ Nurse  □ Other health/healthcare profession  □ Supermarket employee – other retail food store employee  □ Employee in public transportation |
| **Α11. Which is your current employment status now that the measures to prevent the spread of the novel coronavirus into effect/in progress?** | □ Suspension of contract/employment contract on hold  □ Work from home  □ Alternative work arrangements/flexible working options (rota)  I am going to work by turns/alternately/in rotation  □ I go to work as usual  □ I was not employed before and I am not working now/ I was neither employed before nor am I now |
| **Α12.α Were you diagnosed with covid-19?** | □ Yes  □ No |
| **A12.β If yes, did you need to hospitalize?** | □ Yes  □ No |
| **A13. Was any member from your immediate family environment diagnosed with covid-19?**  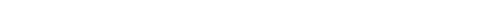 | □ Yes  □ No |

| **Β. Knowledge towards COVID-19** | |
| --- | --- |
| **B1. Which are the most common symptoms of covid-19? (answer with yes, no, I don’t know)** | Yes No I do not know  □ □ □ Gastrointestinal symptoms (e.g. vomiting, diarrhea)  □ □ □ Cough  □ □ □ Shortness of breath  □ □ □ Cough  □ □ □ Myalgia (muscle and skeletal pain)  □ Other (Define) . . . |
| **Β2. With which of the following ways is the novel coronavirus transmitted? (answer with yes, no, I don’t know)** | Yes No I do not know  □ □ □ By air  □ □ □ By air conditioning  □ □ □ By droplets from human to human  □ □ □ By contaminated surfaces  □ □ □ By animals/mosquitos  □ □ □ By food products  □ Other (Define) . . . |
| **Β3. In case you develop symptoms of the novel coronavirus, which is the first thing you will do?**  **(Answer with yes, no, I don’t know)** | Yes No I do not know  □ □ □ Contact the National Public Health Organization  □ □ □ Contact my doctor  □ □ □ Visit a coronavirus reference hospital  □ □ □ Isolate from the rest of my family  □ Other . . . |
| **Β4. Which of the following are considered measures to prevent the spread of the novel coronavirus? (Answer with yes, no, I don’t know)** | Yes No I do not know  □ □ □ Use of gloves  □ □ □ Use of mask  □ □ □ Regular handwashing with soap/alcoholic solution  □ □ □ Avoiding touching your face with your hands  □ □ □ Avoiding contact with animals  □ □ □ Physical distancing (keeping minimum 2 meters between you and others)  □ □ □ Homeopathic remedies  □ □ □ Getting the flu vaccine  □ □ □ Using antibiotics  □ □ □ Regular exercise & healthy diet |
| **Β5. Which of the following is the most appropriate way of hand washing with soap and water? (Choose one answer)** | □ Washing of hands for 5’’ with soap and cold water  □ Washing of hands with soap and warm water  □ Washing of hands for at least 20’’ with soap and water  □ Washing all parts of the hands with foam for 20’’ and dry  □ I don’t know |
| **Β6. Is handwashing with antiseptic/alcoholic solution better than soap and water?** | □ Yes  □ No  □ I don’t know |

| **C. Sources of information about COVID-19** | |
| --- | --- |
| **C1. Which source of information do you use more often regarding the current developments of the COVID-19 pandemic: (choose one answer)** | □ Social media (Facebook, twitter etc.)  □ Official national and state actors’ websites (national organization of public health, ministries etc.)  □ Mass Media (radio, television, newspapers, etc.)  □ Internet  □ Acquaintances and friends  □ Other |
| **D1. Attitudes towards COVID-19** | |
| **D1. Do you believe that the novel coronavirus was developed by humans in laboratories?** | □ Yes  □ No  □ I do not know/I do not answer |
| **D2. How infectious is the novel coronavirus compared to the flu virus H1N1?** | □ Extremely likely  □ Very likely  □ Somewhat likely  □ Slightly likely  □ Not likely at all |
| **D3. How fatal is the novel coronavirus compared to the flu virus H1N1?** | □ Extremely likely  □ Very likely  □ Somewhat likely  □ Slightly likely  □ Not likely at all |
| **D4. When do you think that the spread of the novel coronavirus will be mitigated? (Choose the answer that represents you the most)** | □ when a large percentage of the population has become infected (herd immunity)  □ When the vaccine is developed  □When treatment is found  □ I don’t know/ I don’t answer |
| **D5. Which of the following characteristics of the novel coronavirus infection stresses you out the most? Choose one answer** | □ That is highly contagious  □ That it can cause death  □ That there is no current treatment for the novel coronavirus  □ That there is no vaccine for the novel coronavirus  □ That the actual number of the people infected is unknown  □ I don’t know/ I don’t answer |
| **D6. How likely do you think it is to have other waves of coronavirus outbreaks in our country?** | □ Extremely likely  □ Very likely  □ Somewhat likely  □ Slightly likely  □ Not likely at all  □ I don’t know/ I don’t answer |
| **D7. To what extent do you believe that this pandemic will influence you as far as compliance with preventative measures for infection is concerned in the future?** | □ Strongly Agree  □ Agree  □ Mutual/Neither Agree nor Disagree  □ Disagree  □ Strongly Disagree |
| **D8. To what extent do you believe that this pandemic will positively influence your attitude towards vaccination generally?** | □ Extremely likely  □ Very likely  □ Somewhat likely  □ Not likely at all  □ I don’t know/ I don’t answer |
| **Ε. Attitudes and Behaviors of Social Distancing** | |
| **Ε1. Where you in the process of social distancing before the government measures were applied?** | □ Yes  □ No |
| **Ε2. If yes, to what extent was your decision influenced by each of the following reasons?**  □ Fear for my life  □ Fear of getting infected and transmitting it to people in my close environment who belong to vulnerable groups  □To contribute to minimizing the spread of the virus to the community  □ To avoid overloading and/or collapsing of the healthcare system | □ Very much  □ Much  □Not so much  □A little  □Not at all  □ I don’t know/ I don’t answer |
| **Ε3. How many times the past week have you exiting the house for each of the following reasons?**  □ Going to the pharmacy or doctor visit  □ Going to the supermarket or any other essential goods store  □ Going to the bank  □ Helping people in need  □ Going to a ceremony (e.g. funeral, wedding, baptism)  □ Exercising outside or walking a pet  □ Going to work | □ Once a week  □ 2-3 times a week  □ 4-6 times a week  □ Once a day  □ > once a day  □ None |
| **Ε4. In average how many times do you wash your hands every day?** | □ Less than 5  □ 5 to 10  □ 10 το 20  □ I do not know |
| **Ε5. If there was a vaccine available for the novel coronavirus would you do it?** | □ Yes  □ No  □ I do not know/ I do not answer |
| **Ε6. Did you do the seasonal flu vaccine this year?** | □ Yes  □ No  □ I do not know/ I do not answer |
| **Ε7. Will you do it next year?** | □ Yes  □ No  □ I do not know/ I do not answer |
| **Ε8. During the pandemic did you avoid going to a doctor or a hospital for a severe or chronic health problem?** | □ Yes  □ No |
