# Supplementary material for: Willingness of Greek general population to get a COVID-19 vaccine
Source: Glob Health Res Policy. 2021 Jan 29;6:3. doi: 10.1186/s41256-021-00188-1 (PMC7843240; doi:10.1186/s41256-021-00188-1)
Supplement: Supplementary file 2 — Additional file 2. [file 41256_2021_188_MOESM2_ESM.docx]

**Additional file 2**

**Correct Answers for knowledge score:**

Which are the most common symptoms of covid-19? (answer with yes, no, I don’t know): YES at all these symptoms: Gastrointestinal symptoms, Cough, Shortness of breath, Fever, Myalgia (5 points)

With which of the following ways is the novel coronavirus transmitted? (answer with yes, no, I don’t know): YES by air, by droplets from human to human, by contaminated surfaces, NOT from animals/mosquitos and food products (5 points)

In case you develop symptoms of the novel coronavirus, which is the first thing you will do?

(Answer with yes, no, I don’t know): contact EODY or contact my doctor or isolate from family, NOT visiting reference hospital (1 point)

Which of the following are considered measures to prevent the spread of the novel coronavirus? (Answer with yes, no, I don’t know): Use of gloves, Use of mask, Regular handwashing with soap/alcoholic solution, Avoiding touching your face with your hands, Physical distancing (2m), NOT avoiding contact with animals, homeopathic remedies, flu vaccine, use of abs and healthy diet (10 points)

Is handwashing with antiseptic/alcoholic solution better than soap and water?:ABHR better than soap and water (1point)

Which of the following is the most appropriate way of hand washing with soap and water? (Choose one answer): Washing all parts of the hands with foam for 20’’ and dry (1point)
